# Endothelial Heparan Sulfate Mediates Hepatic Neutrophil Trafficking and Injury during Staphylococcus aureus Sepsis

**DOI:** 10.1128/mBio.01181-21

**Published:** 2021-09-21

**Authors:** Gregory J. Golden, Alejandro Gómez Toledo, Alex Marki, James T. Sorrentino, Claire Morris, Raquel J. Riley, Charlotte Spliid, Qiongyu Chen, Ingrid Cornax, Nathan E. Lewis, Nissi Varki, Dzung Le, Johan Malmström, Christofer Karlsson, Klaus Ley, Victor Nizet, Jeffrey D. Esko

**Affiliations:** a Department of Cellular and Molecular Medicine, University of California, San Diego, La Jolla, California, USA; b Department of Clinical Sciences, Division of Infection Medicine, Lund University, Lund, Sweden; c La Jolla Institute for Allergy and Immunology, San Diego, California, USA; d Department of Bioengineering, University of California, San Diego, La Jolla, California, USA; e Bioinformatics and Systems Biology Graduate Program, University of California, San Diego, La Jolla, California, USA; f Department of Pathology, University of California, San Diego, La Jolla, California, USA; g Department of Pediatrics, University of California, San Diego, La Jolla, California, USA; h Glycobiology Research and Training Center, University of California, San Diego, La Jolla, California, USA; i Novo Nordisk Foundation Center for Biosustainability, University of California, San Diego, La Jolla, California, USA; j Skaggs School of Pharmacy and Pharmaceutical Sciences, University of California, San Diego, La Jolla, California, USA; University of North Carolina at Chapel Hill; Georgia Institute of Technology School of Biological Sciences

**Keywords:** *Staphylococcus aureus*, heparan sulfate, intravital microscopy, liver, neutrophils, proteomics, sepsis, thrombosis

## Abstract

Hepatic failure is an important risk factor for poor outcome in septic patients. Using a chemical tagging workflow and high-resolution mass spectrometry, we demonstrate that rapid proteome remodeling of the vascular surfaces precedes hepatic damage in a murine model of Staphylococcus aureus sepsis. These early changes include vascular deposition of neutrophil-derived proteins, shedding of vascular receptors, and altered levels of heparin/heparan sulfate-binding factors. Modification of endothelial heparan sulfate, a major component of the vascular glycocalyx, diminishes neutrophil trafficking to the liver and reduces hepatic coagulopathy and organ damage during the systemic inflammatory response to infection. Modifying endothelial heparan sulfate likewise reduces neutrophil trafficking in sterile hepatic injury, reflecting a more general role of heparan sulfate contribution to the modulation of leukocyte behavior during inflammation.

## INTRODUCTION

Sepsis is a life-threatening multiorgan system dysfunction caused by a dysregulated host response to severe infection (1). Exaggerated responses to pathogen invasion by the host vasculature and immune system fuel vascular dysfunction, impairing nutrient delivery to vital organs and subsequent organ failure (1–3). Historically, sepsis treatments aimed at inhibition of systemic inflammation have little clinical success (4, 5). Understanding the mechanistic ties between vascular dysfunction and organ failure in sepsis may lead to improved organ support and better outcomes (5).

Vascular surfaces are covered by a layer of glycolipids, glycoproteins, glycosaminoglycans (GAGs) and proteoglycans, collectively termed the vascular glycocalyx (VGC) (6–8). Sepsis dramatically alters the structural and molecular composition of the VGC, promoting leukocyte adherence, vascular dysfunction, and inflammation (9–13). Remodeling of the VGC during sepsis is partially driven by upregulation of endogenous glycosidases and proteases, resulting in shedding of glycan fragments and protein ectodomains that can fuel dysregulated inflammatory loops by acting as damage-associated molecular patterns (DAMPs) (9, 14, 15).

Given their frontline location, the collective glycoproteome and glycome that make up the VGC help orchestrate vascular homeostasis and immunity. Unfortunately, direct examination of VGC molecular composition has been largely performed in the context of isolated endothelial cells, immortalized cell lines or shed VGC material detected in circulation or urine (16). The absence of the normal architecture of the vascular niche in cultured cells limits the usefulness of *in vitro* models for studying VGC remodeling (17). We recently constructed an organ-specific *in vivo* proteome atlas of the murine VGC and showed that it changes significantly during sepsis induced by the leading human invasive pathogen, Staphylococcus aureus (13). Proteome changes during bacterial challenge were tightly correlated with tissue damage, suggesting a link between vascular dysfunction and organ failure. Whether vascular proteome remodeling precedes tissue damage or is just a consequence of generalized organ failure is an important question that remains to be addressed in this sepsis model system.

S. aureus sepsis leads to pathogen accumulation primarily in the liver (13, 18), where tissue-resident macrophage Kupffer cells filter circulating bacteria (19, 20), stimulating a wave of infiltrating neutrophils in the sinusoids that attempt to clear the infection. Activated neutrophils release neutrophil extracellular traps (NETs), triggering an immunothrombotic response that helps to trap and ensnare bacteria, although at a significant inflammatory cost (18, 21). In sepsis, the hepatic neutrophilic response progresses to a vasculopathic and hypercoagulative syndrome and results in blood vessel occlusion, necrosis, and liver failure (13, 18). Neutrophil trafficking into the liver during nonsterile inflammation partially depends on neutrophil expression of CD44 that tightly binds to sinusoidal hyaluronan (HA), an abundant GAG in the hepatic vascular glycocalyx (18, 22, 23). Notably, neutrophil chemotaxis to the liver during sterile hepatic inflammation is mostly mediated by an integrin-dependent mechanism (23), and understanding which VGC components regulate this neutrophil trafficking might inform pharmacological strategies to protect the organs during sepsis.

Heparan sulfate (HS), a type of sulfated GAG, is a major VGC component that impacts multiple aspects of vascular inflammation and sepsis (24). Endothelial HS participates in the formation of chemokine gradients and chemokine transcytosis that attract leukocytes toward sites of inflammation (25–33) and modulate neutrophil extravasation by interacting with selectins during neutrophil rolling in peripheral tissues (26, 30). Exogenous HS oligosaccharides block neutrophil influx and inflammation in acetaminophen-induced liver failure (34), while intravenous injection of the recombinant HS-binding domain of CXCL9 competes with the endogenous chemokine for VGC binding and blunts neutrophil trafficking (35–37). In this report, we show that proteins that bind HS are significantly enriched in the hepatic VGC during S. aureus sepsis. Genetic modification of the fine structure of endothelial HS diminishes neutrophil trafficking and subsequent pathological thrombosis in the liver vasculature. Together, HS and HA play important roles in neutrophil trafficking in the liver.

## RESULTS

**FIG 1:**
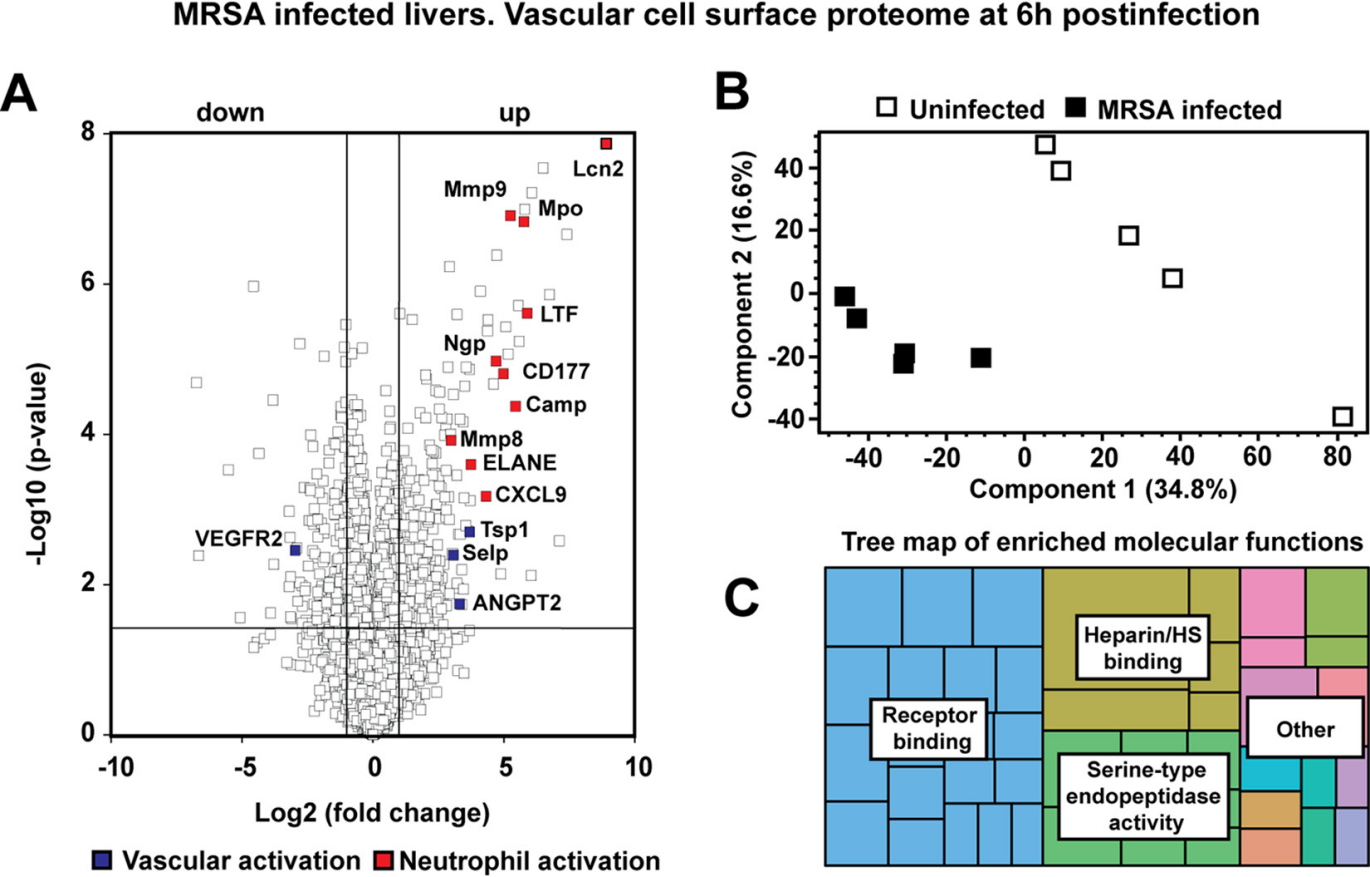
(A) Volcano plot of liver vascular-tagged proteins at 6 h postinfection. The horizontal line indicates the significance *P* value cutoff of 0.05, and the vertical lines refer to the fold change values of −2 and 2. (B) PCA plot of enriched liver vascular-tagged proteins in uninfected and 6-h postinfection samples. (C) Tree map of enriched molecular functions from enriched liver vascular-tagged proteins 6 h postinfection. Major classes of enriched molecular functions are indicated within their respective colored areas.

Previously, we characterized VGC remodeling after S. aureus challenge to ascertain its composition during severe hepatic damage and pathology (13), but these studies were performed at 24 h postinfection. To characterize VGC remodeling during the initial stages of liver inflammation (18), we applied a similar chemical tagging and proteomics workflow to a murine model of S. aureus sepsis 6 h postinfection. Briefly, systemic perfusion of the mouse vasculature using a sulfo-*N*-hydroxysuccinimide (NHS)-biotin solution was performed to tag proteins exposed to the vascular flow and to facilitate downstream streptavidin affinity purification and proteomic characterization (13). The vascular cell surface proteome of the murine liver was significantly altered at this earlier time point (Fig. 1A; Table S1). In total, 5,753 proteins were identified and quantified, of which 647 were significantly changed upon infection. Early signs of inflammation included higher levels of the serum amyloid proteins (Saa1-3) and C-reactive protein, as well as vascular expression of Toll-like receptor 2 (Tlr2), which is known to mediate host responses to Gram-positive bacteria through peptidoglycan and lipoteichoic acid interactions. Neutrophil-derived proteins such as myeloperoxidase (Mpo), neutrophil elastase (ELANE), matrix metallopeptidase 9 (Mmp9), and neutrophil gelatinase-associated lipocalin (Lcn2) were among the most accentuated proteins in our data sets (Fig. 1A), suggesting that neutrophil influx and NET formation start early during infection and precede organ damage. Vascular activation was also indicated by increased P-selectin (Selp), thrombospondin 1 (Tsp1), and angiopoietin 2 (ANGPT2) levels, coupled to decreased presentation of vascular receptors such as the vascular growth factor receptor 2 (VEGFR2). Vascular proteome changes also included alterations in various antimicrobial factors, cytokines, and chemokines. Principal-component analysis (PCA) of the proteome fraction clearly separated infected from uninfected samples (Fig. 1B), and molecular functions for receptor activation and binding, endopeptidase activity, and heparin/HS binding proteins were highly enriched in the hepatic vasculature (Fig. 1C). The latter group is notable since HS is directly involved in many critical receptor-ligand interactions during inflammatory responses to modulate neutrophil trafficking (38).

10.1128/mBio.01181-21.5 TABLE S1 The vascular cell surface proteome of the murine liver; in total, 5,753 proteins were identified and quantified, of which 647 were significantly changed upon infection. Download Table S1, XLSX file, 0.8 MB. Copyright © 2021 Golden et al. 2021 Golden et al. https://creativecommons.org/licenses/by/4.0/ This content is distributed under the terms of the Creative Commons Attribution 4.0 International license.

To examine how vascular HS affects liver pathology during S. aureus infection, the bifunctional HS *N-*deacetylase/*N-*sulfotransferase 1 (*Ndst1*) gene was inactivated by driving Cre recombinase expression in endothelial and myeloid cells through the *Tie2* (*Tek*) promoter (*Ndst1^f/f^Tie2Cre*) (39). We previously reported that inactivation of *Ndst1* results in significantly undersulfated HS in endothelial cells, thereby disrupting native HS-protein interactions and biological responses (26, 40). Partial desulfation of the chains occurs because most tissues also express *Ndst2* (41). Removal of both genes or systemic inactivation of *Ndst1* leads to embryonic or perinatal lethality, respectively, thus requiring the use of conditional mutants (42–44).

**FIG 2:**
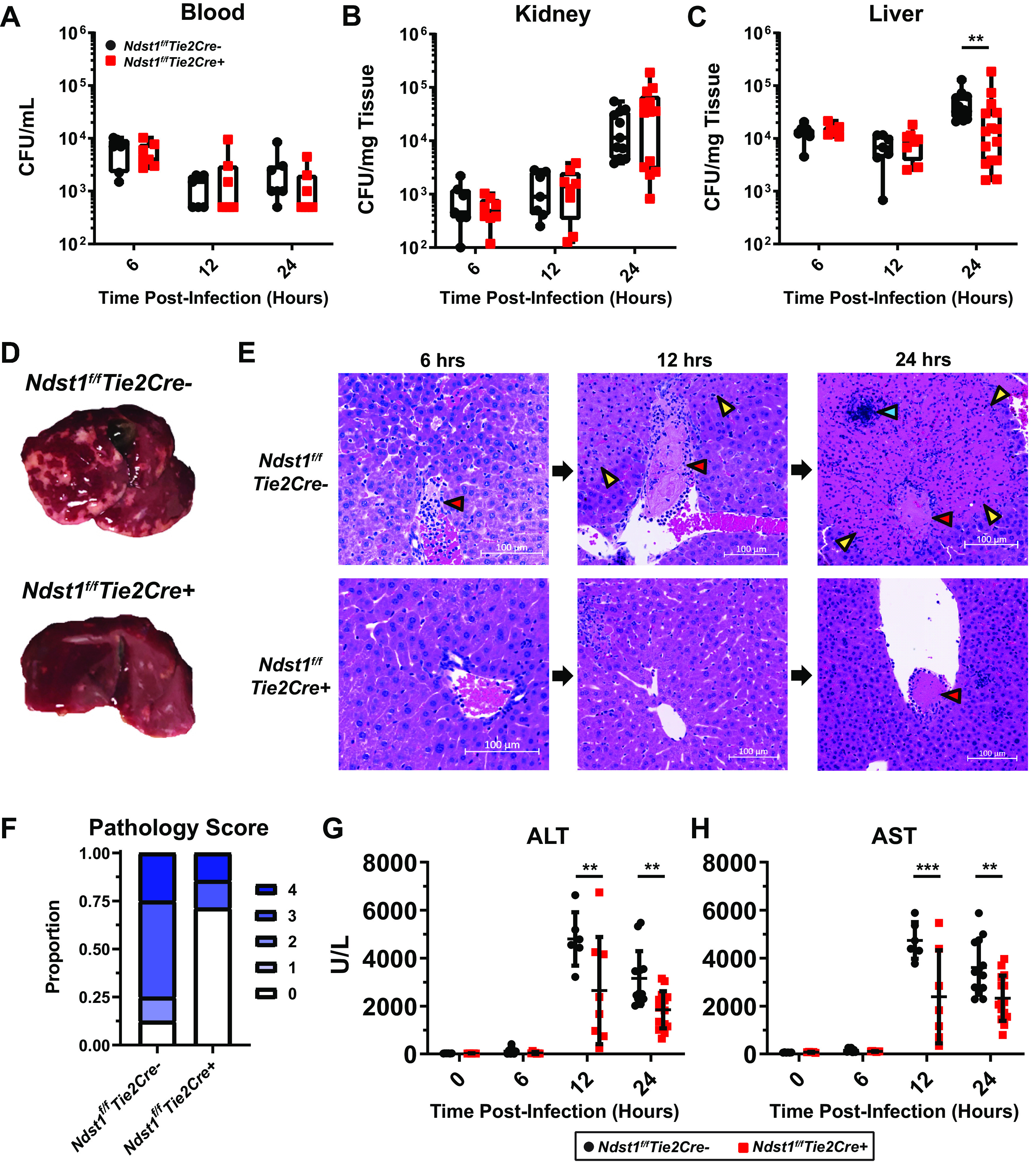
(A) Blood, (B) kidney, and (C) liver CFU from the indicated time points. *n* ≥ 7 per group. Boxes indicate min, max, and quartile datapoints. **, *P < *0.01 as determined by a Mann-Whitney U test due to nonnormal distribution of data points. (D) Representative liver gross pathology 24 h postinfection. Pale regions demark areas of necrosis. (E) Representative liver histopathology at the indicated time points postinfection. Neutrophil-surrounded thrombi (red arrows) occlude vessels and correspond to regions of coagulative necrosis (hepatic infarction, yellow arrow,) that may contain bacterial colonies (blue arrow). Scale bars = 100 μm. (F) Liver histopathology scores 24 h postinfection. *n* = 7 to 8 per group. Scores were assigned from 0 = no pathology to 4 = severe inflammation and necrosis. (G) Serum ALT and AST levels across the indicated time points. *n* ≥ 7 per group. Errors bars represent the mean ± the standard error of the mean (SEM). **, *P < 0*.01; ***, *P* < 0.001 as determined by a 2-way analysis of variance (ANOVA) with Sidak’s multiple-comparison test between genotypes.

S. aureus sepsis was induced in *Ndst1^f/f^Tie2Cre+* and wild-type (*Ndst1^f/f^Tie2Cre–*) litter mate control mice, and CFU were enumerated in several tissues at multiple time points. While about 50 to 75% of the animals succumb to this infectious challenge within 48 h, no differences in general behavior or outward signs of distress between mutant and wild-type mice were noted in the first 12 h. S. aureus bacteremia and organ colonization were readily detectable at 6 h postinfection (Fig. 2A to C). Consistent with previous findings, the liver harbored the highest CFU burden early in infection (6 h; Fig. 2C) (13, 18). Although median levels of bacteremia fluctuated only slightly over time (Fig. 2A), the median organ CFU counts increased 24-fold in the kidney and 5-fold in the liver by 24 h postinfection in *Ndst1^f/f^Tie2Cre–* animals. In contrast, *Ndst1^f/f^Tie2Cre+* mice did not exhibit such a dramatic increase in liver CFU over the time course (≤2-fold on average) (Fig. 2C). *Ndst1^f/f^Tie2Cre–* and *Ndst1^f/f^Tie2Cre+* mice had similar levels of bacteremia (Fig. 2A) and similar increases in kidney CFU (Fig. 2B), indicating that the overall degree of HS sulfation selectively impacts S. aureus bacterial load in the liver.

Following S. aureus challenge, the liver develops significant coagulopathy that occludes its vasculature, leading to subsequent grossly apparent necrosis of the surrounding parenchyma (Fig. 2D) (13). In *Ndst1^f/f^Tie2Cre–* mice, liver thromboses began to develop early after infection with influxing neutrophils, consistent with the findings from the vascular proteomic analysis (Fig. 2E) (45, 46). By 12 h postinfection, thrombus formation was readily apparent (Fig. 2E, red arrowheads), with neutrophils surrounding the thrombi, and evidence of hepatic necrosis (Fig. 2E, yellow arrowheads). By 24 h postinfection, many vessels were completely occluded with corresponding areas of coagulative necrosis and intralesional bacterial colonies (Fig. 2E, blue arrowhead), paralleling the large increase in S. aureus CFU at this time point (Fig. 2C). Strikingly, *Ndst1^f/f^Tie2Cre+* mice exhibited fewer grossly visible necrotic lesions 24 h postinfection (Fig. 2D). Scoring of liver histopathology showed reduced damage at 24 h postinfection in *Ndst1^f/f^Tie2Cre+* mice (Fig. 2E and F). Serum markers of liver damage coincided with liver pathology. *Ndst1^f/f^Tie2Cre–* mice had large increases in serum alanine aminotransferase (ALT) and aspartate aminotransferase (AST) at 12 and 24 h postinfection (Fig. 2G and H), whereas ALT/AST elevations were significantly smaller in *Ndst1^f/f^Tie2Cre+* mice (Fig. 2G and H). Reducing sulfation of endothelial HS did not affect the levels of serum markers of kidney damage, again indicative of selective activity toward the liver (Fig. S1A).

10.1128/mBio.01181-21.6 FIG S1 (A) Serum blood urea nitrogen levels across the indicated time points postinfection. *n* = 7 per group. Error bars represent the mean ± SEM. (B) Serum ALT and (C) AST levels from 24 h postinfection. *n* = 9 to 12 mice per group. Errors bars represent the mean ± SEM. (D) Liver CFU from 24 h postinfection. *n* = 9 to 12 per group. Boxes indicate min, max, and quartile datapoints. Download FIG S1, JPG file, 1.3 MB. Copyright © 2021 Golden et al. 2021 Golden et al. https://creativecommons.org/licenses/by/4.0/ This content is distributed under the terms of the Creative Commons Attribution 4.0 International license.

Tie2-driven gene inactivation can result in recombination of “floxed” genes in the hematopoietic compartment in addition to the endothelium (47), which is an important consideration, as both myeloid and platelet lineages contribute to S. aureus-induced liver damage (48). To evaluate the potential contribution of myeloid- and platelet-specific *Ndst1* inactivation to the liver phenotype, *Ndst1* was selectively inactivated using a combination of *LysMCre* and *PF4Cre* drivers (*Ndst1^f/f^LysM/PF4Cre*). *Ndst1^f/f^LysM/PF4Cre+* mice had comparable levels of liver CFU and plasma ALT and AST as *Ndst1^f/f^LysM/PF4Cre–* mice at 24 h postinfection (Fig. S1B to D), indicating that reduction of endothelial HS sulfation, and not of myeloid HS, drives the hepatic phenotype in the model.

**FIG 3:**
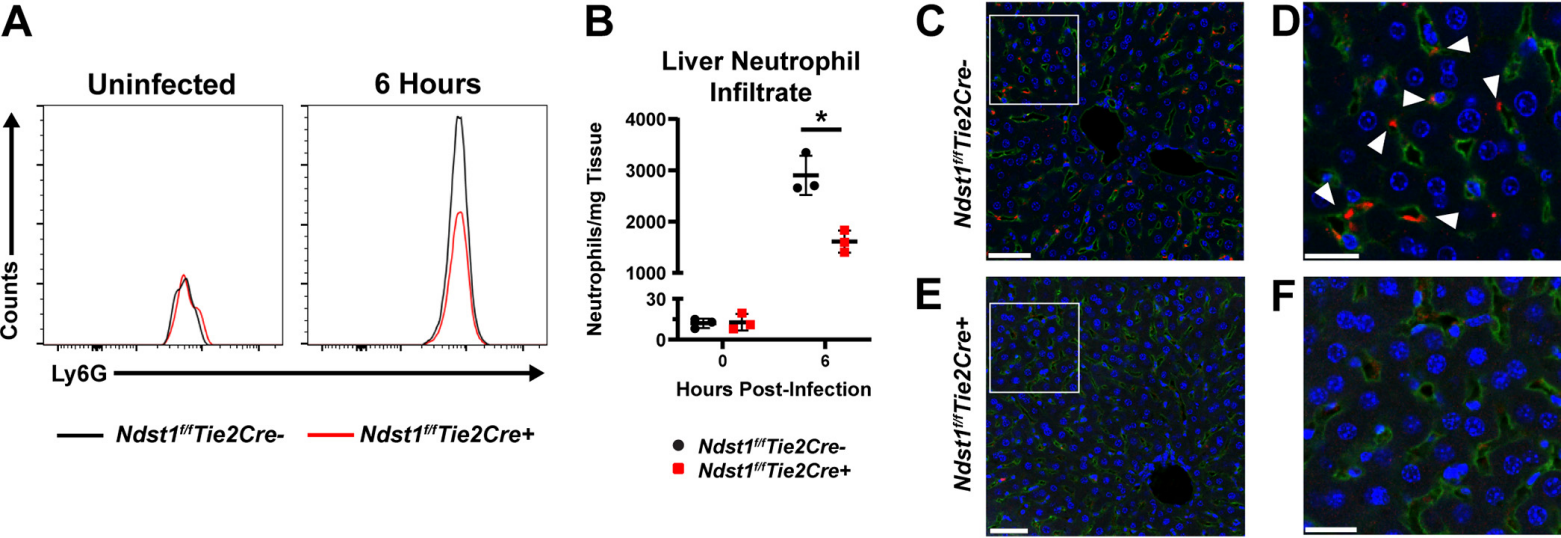
(A) Representative flow distributions of neutrophils (Ly6G+) from uninfected and 6-h postinfection livers. (B) Neutrophil counts per mg of liver at the indicated time points, as determined by flow cytometry. *n* = 3 per genotype, and is representative of 3 independent experiments. *, *P < *0.05. (C to F) Representative immunofluorescent images of liver 6 h postinfection. Green, CD68 (macrophages and endothelial cells); red, MPO; blue, DAPI. (C) Representative immunofluorescent image of *Ndst1^f/f^Tie2Cre–* liver. (D) Magnified area indicated in panel C. Arrows indicate sinusoidal MPO. (E) Representative immunofluorescent image of *Ndst1^f/f^Tie2Cre+* liver. (F) Magnified area indicated in panel E. In panels C and E, scale bars = 100 μm, and in panels D and F, scale bars = 25 μm.

Neutrophilic infiltration is required for the hepatic damage characteristic of S. aureus sepsis (18). Neutrophils bind to HA in the sinusoidal glycocalyx, leading to their sequestration to the liver vasculature during infection, promoting inflammation and vascular coagulation (18, 22). In contrast, neutrophil trafficking in the peripheral vasculature is modulated by endothelial HS (26). To examine if endothelial HS also participates in hepatic leukocyte trafficking during sepsis, neutrophils and monocytes were quantitated by flow cytometry at 6 h postinfection (Fig. S2A). A marked increase in neutrophil infiltration into the liver occurred in *Ndst1^f/f^Tie2Cre–* mice, whereas the extent of infiltration in the livers of *Ndst1^f/f^Tie2Cre+* mice was reduced ∼2-fold (Fig. 3A and B), consistent with trafficking deficiencies previously observed in the skin of these mice using air pouch models (26). Monocytes also infiltrated the liver, but the extent of infiltration did not differ between mutant and wild-type mice (Fig. S2B). Circulating neutrophil and monocyte counts were also comparable in mutant and wild-type mice, in both uninfected and infected animals (Fig. S2C and D); thus, differences in liver neutrophil counts did not derive from differences in circulating neutrophil and monocyte abundance. Immunofluorescent staining of liver sections revealed cells in the sinusoids of *Ndst1^f/f^Tie2Cre–* mice that stained strongly for myeloperoxidase (MPO) (Fig. 3C and D), consistent with infiltration and sequestration of neutrophils in the liver sinusoids (18). MPO-stained cells (neutrophils) were not as prominent in *Ndst1^f/f^Tie2Cre+* liver, indicative of fewer infiltrating neutrophils (Fig. 3E and F). This reduction of neutrophil infiltration likely spares *Ndst1^f/f^Tie2Cre*+ mice from severe hepatoxicity (Fig. 2), similar to how blocking neutrophil infiltration through CD44 inactivation reduces S. aureus-induced hepatic damage (18).

10.1128/mBio.01181-21.7 FIG S2 (A) Representative flow cytometry workflow of CD45^+^CD11b^+^Ly6G^+^ neutrophil and CD45^+^CD11b^+^Ly6C^hi^ monocyte infiltrate in the liver. (B) Monocyte counts in the liver as counted by flow cytometry at the indicated time points, representative of 3 experiments. (C) Neutrophil and (D) monocyte counts in the blood in healthy or 18-h postinfection mice. Download FIG S2, JPG file, 1.8 MB. Copyright © 2021 Golden et al. 2021 Golden et al. https://creativecommons.org/licenses/by/4.0/ This content is distributed under the terms of the Creative Commons Attribution 4.0 International license.

**FIG 4:**
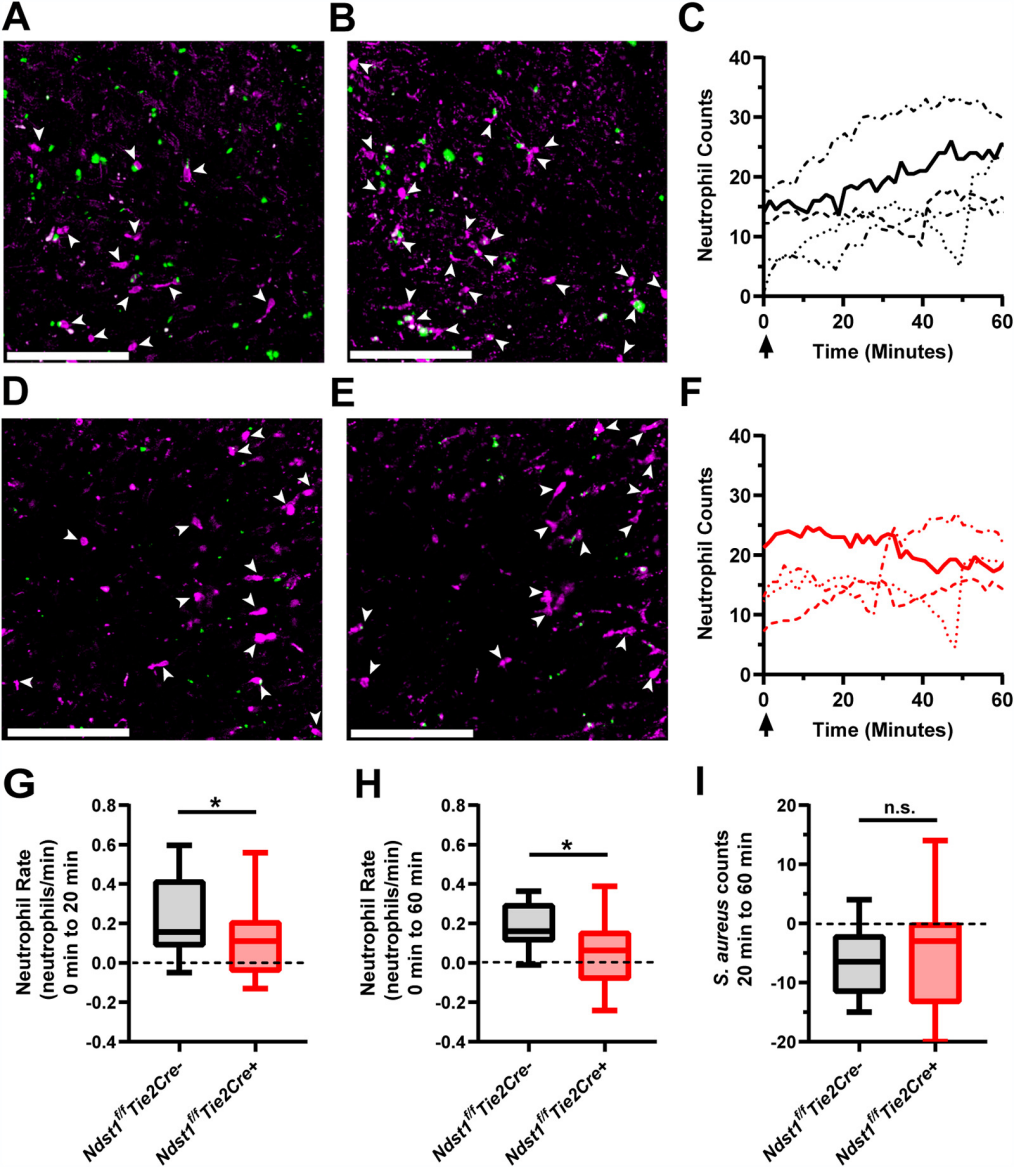
(A) Representative intravital image of liver from an *Ndst1^f/f^Tie2Cre–* mouse immediately post-i.v. infection of S. aureus (green) and (B) 60 min postinfection. Panels A and B are the same field of view (FOV). Neutrophils are demarked by white arrows. Ly6G antibody (magenta) strongly labels neutrophils, with some nonspecific staining of the tissue. Scale bars = 200 μm. (C) Liver neutrophil counts over time as tracked by IVM in *Ndst1^f/f^Tie2Cre–* mice. Each line represents counts from 4 FOV per mouse, with single data points determined by averaging neutrophil counts from the 4 FOV. The black arrow denotes the time of S. aureus injection. (D and E) Representative intravital image of liver from an *Ndst1^f/f^Tie2Cre+* mouse (D) immediately postinfection and (E) 60 min postinfection. All colors and markings are the same as in panels A and B. (F) Liver neutrophil counts over time as tracked by IVM in *Ndst1^f/f^Tie2Cre+* mice. Data were generated as in panel C. (G and H) Rates of neutrophil recruitment for (G) 20 min and (H) 60 min postinfection as determined by IVM. (I) Change in S. aureus count from 20 min to 60 min postinfection. For panels G to I, each data point represents 1 FOV, with *n* = 4 FOV per mouse and *n* = 4 to 5 mice per genotype. Box and whisker plots are min to max with quartiles demarked. *, *P* < 0.05.

Finally, we used intravital microscopy (IVM) to measure the kinetics of neutrophil trafficking immediately following infection. GFP-positive S. aureus injected intravenously (i.v.) was visible and adherent in the liver sinusoids within seconds of injection (Video S1). By 20 min postinfection, bacteria ceased to accumulate in the liver (Video S1). The majority of bacteria adhered to resident Kupffer cells (49) and neutrophils. Some neutrophils appeared to phagocytose S. aureus and continued to traffic through the liver vasculature (Video S2). *Ndst1^f/f^Tie2Cre–* mice exhibited higher neutrophil counts a few minutes after injection that increased over the first hour of infection (Fig. 4A to C; Video S3). In contrast, *Ndst1^f/f^Tie2Cre+* mice showed only a marginal increase in neutrophil counts during the first hour after infection (Fig. 4D to F; Video 3). The rate of neutrophil recruitment in *Ndst1^f/f^Tie2Cre+* mice was ∼50% the rate observed in *Ndst1^f/f^Tie2Cre–* mice (Fig. 4G and H), consistent with the reduced neutrophil counts observed at 6 h postinfection by flow cytometry (Fig. 3B). Initial S. aureus counts were similar and declined at the same rate in both mutant and wild-type animals (Fig. 4I), indicating that initial bacterial burden does not depend upon vascular HS status.

10.1128/mBio.01181-21.1 VIDEO S1 Representative video of the first 20 min postinfection with S. aureus. First, 5 × 10^7^ CFU S. aureus expressing GFP were injected i.v. immediately after imaging initiation in the liver. Neutrophils (strong magenta) are present in the sinusoids (weak magenta). Imaging was collected using time-lapse intravital confocal microscopy. Scale bar = 100 μm. Images were collected every 90 s with a display rate of 12 frames/s. Download Movie S1, AVI file, 0.9 MB. Copyright © 2021 Golden et al. 2021 Golden et al. https://creativecommons.org/licenses/by/4.0/ This content is distributed under the terms of the Creative Commons Attribution 4.0 International license.

10.1128/mBio.01181-21.2 VIDEO S2 A neutrophil (strong magenta) in the liver interacting with circulating S. aureus, as highlighted by the white arrow. The neutrophil continues to migrate after interaction and carries the bacteria during migration. Bacterial injection, imaging parameters, and display parameters are the same as in Video S1. Download Movie S2, AVI file, 0.7 MB. Copyright © 2021 Golden et al. 2021 Golden et al. https://creativecommons.org/licenses/by/4.0/ This content is distributed under the terms of the Creative Commons Attribution 4.0 International license.

10.1128/mBio.01181-21.3 VIDEO S3 Representative video of *Ndst1^f/f^Tie2Cre–* (part 1) and *Ndst1^f/f^Tie2Cre+* (part 2) livers in the first 60 min postinfection with S. aureus. Bacterial injection, imaging parameters, and display parameters are the same as in Video S1. Download Movie S3, AVI file, 1.3 MB. Copyright © 2021 Golden et al. 2021 Golden et al. https://creativecommons.org/licenses/by/4.0/ This content is distributed under the terms of the Creative Commons Attribution 4.0 International license.

**FIG 5:**
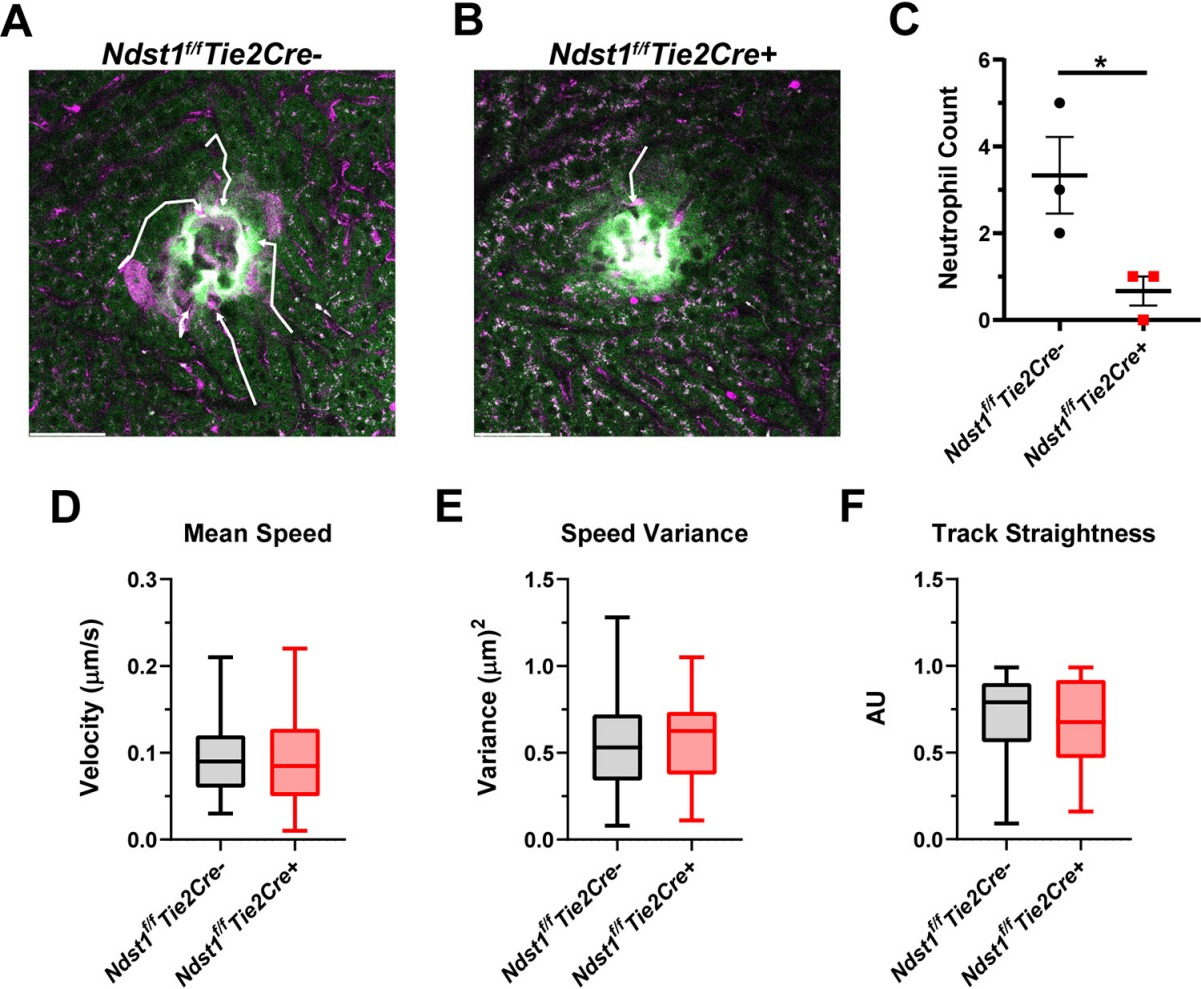
(A and B) Representative intravital image of (A) *Ndst1^f/f^Tie2Cre–* and (B) *Ndst1^f/f^Tie2Cre+* livers 20 min after sterile injury. Ly6G+ neutrophils (magenta) that trafficked to the injury are denoted by white arrows. Autofluorescence was used to visualize the injury (green) and surrounding hepatocytes (light green). (C) Number of neutrophils that reach the injury within 20 min of insult. Each point represents 1 FOV in 1 mouse. Each mouse is an independent experiment. *, *P* < 0.05. (D) Mean speed, (E) speed variance, and (F) track straightness of neutrophils that reached the sterile wound. For panels D to F, each data point represents a single neutrophil that reached the wound over a 3-h timespan with *n* ≥ 36 with data pooled across 3 mice per genotype. For panels D to F, box and whisker plots are min to max with quartiles demarked.

Neutrophil trafficking during sterile inflammation in the liver is based upon an integrin-dependent mechanism and not on HA (23). To determine if vascular HS also modulates neutrophil trafficking in sterile inflammation, a focal sterile injury was applied to the livers of *Ndst1^f/f^Tie2Cre* mice with a two-photon laser, and neutrophils were tracked using IVM. Immediately following sterile injury, neutrophils were attracted to the injury site in *Ndst1^f/f^Tie2Cre–* mice (Fig. 5A and C; Video S4), but neutrophil trafficking was blunted in *Ndst1^f/f^Tie2Cre+* mice (Fig. 5B and C; Video S4). Endothelial HS acts as a ligand for L-selectin during neutrophil rolling (26, 30). However, altering endothelial HS did not change trafficking characteristics such as mean speed, speed variance, or track straightness during sterile liver inflammation, suggesting that L-selectin-mediated tethering might not be affected in the *Ndst1^f/f^Tie2Cre*+ mutant (Fig. 5D to G).

10.1128/mBio.01181-21.4 VIDEO S4 Representative video of *Ndst1^f/f^Tie2Cre–* (part 1) and *Ndst1^f/f^Tie2Cre+* (part 2) livers immediately prior to and in the first 25 min postinjury. Neutrophils (strong magenta) are present in the sinusoids (weak magenta). Autofluorescence (green) was used to visualize tissue structure and the sterile injury. Imaging was collected using time-lapse intravital confocal microscopy. Scale bar = 100 μm. Preinjury, images were collected every second with a display rate of 10 frames/s. Postinjury, images were collected every 60 s with a display rate of 12 frames/s. Download Movie S4, AVI file, 4.6 MB. Copyright © 2021 Golden et al. 2021 Golden et al. https://creativecommons.org/licenses/by/4.0/ This content is distributed under the terms of the Creative Commons Attribution 4.0 International license.

## DISCUSSION

In summary, our findings show that S. aureus induces dramatic compositional changes in the liver VGC within 6 h of infection, consistent with altered neutrophil infiltration, endothelial activation, and alterations in the vascular HS interactome. Modifying the fine structure of HS in the VGC mitigates neutrophil infiltration and reduces subsequent vascular inflammation and hepatic damage. HS sulfation does not appear to impact the initial colonization of hepatic tissues by S. aureus, as bacterial burden in the liver was the same immediately after infection (Fig. 4I). However, at 24 h postinfection, *Ndst1^f/f^Tie2Cre+* mice exhibited lower S. aureus burden in the liver (Fig. 2B). In *Ndst1^f/f^Tie2Cre–* mice, increased neutrophil infiltration in the liver drives vascular coagulopathy, leading to occlusion of the blood vessels and subsequent tissue necrosis, which could potentially create a more conducive environment for bacterial growth. Further studies are needed to support this hypothesis, among other possible inflammatory changes that may occur in the septic liver after vascular HS modification. For example, HS binds to histones in NETs, which are both proinflammatory and promote coagulation (18, 45, 50). Importantly, our work suggests that vascular HS participates in the attraction of neutrophils to the liver in bacterial sepsis and in sterile inflammation. However, we have found no connection between the liver phenotype and infection-induced mortality in *Ndst1Tie2Cre*+ mice. Many factors contribute to the lethality of sepsis, and we suspect that lethality may be associated with stress and failure in nonhepatic systems later in infection. Our studies provide insight into early changes in the VGC and the role of the VGC in acute hepatic inflammation and coagulopathy.

Additional studies are needed to understand the mechanism of HS-dependent neutrophil recruitment in the liver. Although *Ndst1^f/f^Tie2Cre+* mice have less thrombotic lesions in the liver during S. aureus infection (Fig. 2E), we hypothesize that the phenotype is downstream of reduced neutrophil influx, as intravascular coagulation parameters are unchanged in *Ndst1^f/f^Tie2Cre+* mice (26). In peripheral tissues, endothelial HS appears to play three roles in neutrophil recruitment. First, endothelial HS can bind and sequester chemokines, facilitating the formation of chemotactic gradients (38); heparan sulfate proteoglycans (HSPGs) are thought to mediate chemokine transfer across the endothelium from sites of inflammation (26, 30), and the HS chains bind to L-selectin on neutrophils (26, 30). Thus, we can now extend these earlier studies that showed that altering the structure of HS decreased neutrophil infiltration induced by acute inflammatory challenges to the liver. One would expect endothelial HS modulation would have similar effects in all organs during S. aureus infection, but there are probably other organ-specific responses that modulate infiltration of inflammatory cells, such as specific chemokines and adhesive factors. The acute nature of the infection and infiltration, and the liver’s efficient sequestration of a vast majority of circulating S. aureus (18), likely amplifies any inflammatory defects that occur in the liver. Further studies will seek to delve deeper into molecular mechanism(s) by which HS mediates neutrophil migration in the liver in both sterile and nonsterile injuries and how these systems are coordinated with the CD44-HA axis.

Previous studies have shown that Kupffer cells lining the sinusoids quickly filter S. aureus from circulation, leading to an influx of neutrophils in a CD44-dependent mechanism (18). Our work adds additional insight into the bacterial filtration and neutrophil trafficking mechanism. Neutrophils present in the liver sinusoids also directly sequester circulating S. aureus and continue to traffic with captured bacteria, indicating that bacterial filtration from the bloodstream is not solely executed by Kupffer cells. Others studies have shown that platelets bundle bacteria in the bloodstream and boost the activity of professional phagocytes (51), although it is unclear if platelets execute this function in the context of the liver. Nonetheless, filtration of S. aureus from circulation is extremely rapid and occurs within minutes of bacterial injection. Within this time frame, signaling modulated by endothelial HS initiates liver neutrophil accumulation. Although previous studies have indicated that liver neutrophil accumulation occurs 4 h after systemic S. aureus infection (18), the HS-mediated chemotactic signal is functional within minutes of infection. As chemokines are bound to HS and can rapidly signal to neutrophils (10, 38), future studies will focus on HS-dependent chemokine signaling in the liver during S. aureus infection.

During S. aureus sepsis, major organs undergo dramatic vascular glycocalyx changes that reflect the organ microenvironment (13). At 24 h postinfection, the liver vasculature is enriched with HA recognition and remodeling proteins (13). However, at 6 h postinfection, the liver vasculature signature is dominated by neutrophil proteins and endothelial activation markers, indicating a temporal dynamic to VGC composition throughout S. aureus sepsis host responses. Characterizing VGC dynamics in critical organs throughout septic responses would yield valuable information about the vascular environment at different stages of the disease. The VGC compositional analyses completed thus far give critical insight into the liver vascular environment during S. aureus sepsis. Expansion of these temporal studies across multiple organs and sepsis-causing agents would give insight into why certain organs are prone to failure in sepsis, as the glycocalyx is critical to vascular function (16). Understanding how the organ-vascular interface changes in sepsis could provide critical information on how certain organs fail and how to support organs prone to failure, thus promoting positive outcomes in sepsis. Although we demonstrated that proteins associated with the liver VGC change during early sepsis and that endothelial HS plays a role in subsequent hepatic inflammation, other components of the VGC may have changed as well in response to the alteration in HS sulfation. Future efforts will focus on increasing the molecular and temporal resolution of the VGC in sepsis and generating diagnostics and theragnostics that provide information about the health of the organ-vascular interface during sepsis.

## MATERIALS AND METHODS

### Bacterial strains and preparation.

Staphylococcus aureus (strain USA300 TCH1516) was originally isolated from an outbreak in Houston, Texas, and caused severe invasive disease in adolescents (52). S. aureus was routinely grown at 37°C on Todd-Hewitt agar (Difco) or in liquid cultures of Todd-Hewitt broth (THB; Difco) with stirring (200 rpm). Bacteria were inoculated into 5 ml of fresh THB and incubated overnight. An aliquot of the overnight culture (0.4 ml) was inoculated into 6 ml of fresh THB and incubated to an optical density at 600 nm (OD_600_) of 0.4. Bacteria were sedimented by centrifugation, washed twice with phosphate-buffered saline (PBS), and suspended in PBS at 5 × 10^8^ CFU/ml. S. aureus USA300 TCH1516 constitutively expressing green fluorescent protein (GFP) was cultured using the same method.

### Animal studies.

*Ndst1^f/f^* transgenic C57bl/6 mice were crossed with *Tie2Cre* transgenic C57bl/6 mice to generate *Ndst1^f/f^Tie2Cre* mice (26). To generate *Ndst1^f/f^LysM/PF4Cre* mice, B6.129P2-*Lyz2^tm1(cre)Ifo^*/J (LysMCre; Jackson Laboratory) were crossed to *Ndst1^f/f^* transgenic C57BL/6 mice to generate *Ndst1^f/f^LysMCre* mice, and C57BL/6-Tg(Pf4-icre)Q3Rsko/J (PF4Cre; Jackson Laboratory) were crossed to *Ndst1^f/f^* transgenic C57bl/6 mice to generate *Ndst1^f/f^PF4Cre* mice. The *Ndst1^f/f^LysMCr;Pf4Cre* line was generated by crossing *PF4Cre* and LysMCre mice, as described elsewhere (48). C57BL/6 male and female mice (8 to 10 weeks old) were injected i.v. through the retroorbital sinus with 5 × 10^7^ CFU (0.1 ml) S. aureus. At 24 h postinfection, animals were euthanized with isoflurane and immediately processed for sample collection. CFU in the S. aureus inoculum were enumerated by serial dilution on Todd Hewitt agar plates to ensure consistent CFU dosing across experiments. Animals were housed and bred in individual ventilated cages in a specific-pathogen-free background, in vivaria approved by the Association for Assessment and Accreditation of Laboratory Animal Care located in the School of Medicine, UC San Diego. All experiments were performed in accordance with relevant guidelines and regulations following standards and procedures approved by the UC San Diego Institutional Animal Care and Use Committee (protocols S99127 and S00227M) and the La Jolla Institute for Immunology Department of Laboratory Animal Care (protocol AP00001019).

### Blood chemistry and complete blood count.

To collect serum for blood chemistry, blood was collected via cardiac puncture and placed in a procoagulant serum tube (BD Microtainer no. 365967) for 4 h at room temperature. Serum was isolated by centrifugation (2,000 × *g* for 10 min). All samples were frozen before analysis. Blood chemistry parameters were measured on a Cobas 8000 automated chemistry analyzer (Roche) with a general coefficient of variance of <5%. For complete blood count, blood was mixed with a citrate-dextrose solution (1:9 vol/vol) (Millipore Sigma; no. C3821) and analyzed on a Hemavet 950FS multispecies hematology system (Drew Scientific, CT) programmed to mouse settings.

### Histological analysis.

Tissues were submersion-fixed in 10% neutral buffered formalin (Fisher Chemical) for 24 h, followed by 70% ethanol for at least 24 h. The samples were paraffin-embedded and sectioned (3 μm) and stained with hematoxylin/eosin. Sections underwent blinded scoring by a veterinary pathologist to measure liver inflammation and necrosis, with scores ranging from 0 to 4 (4 indicating severe inflammation and necrosis).

### Bacterial CFU counts.

Organs of interest were placed in a 2-ml tube (Sarstedt; no. 72.693.005) containing 1 ml ice cold PBS and 1-mm-diameter zirconia/silica beads (Biospec Products; no. 11079110z). Samples were homogenized using a MagNA Lyzer (Roche) for 2 min at 6,000 rpm. An aliquot of each organ sample was serially diluted in PBS and plated on Todd-Hewitt agar to enumerate CFU.

### Single cell suspension and flow cytometry.

Mice were euthanized with isoflurane and immediately perfused at 7 ml/min for 2 min with ice-cold PBS through the left ventricle, with a small cut made in the right ventricle for drainage of perfusate. The left lobe of the liver was isolated from each mouse and minced with scissors in ice-cold petri dishes. Samples were then resuspended in 5 ml of ice-cold Hanks balanced salt solution (HBSS) with Ca^2+^/Mg^2+^ (Thermo Fisher Scientific; no. 14025092) containing 3 mM CaCl_2_ in a 50-ml conical tube. The tissue was adjusted to 0.3 units/ml of Liberase TL (Roche; no. 5401020001) and 40 units/ml DNase I (Millipore Sigma; no. D4263) and stirred at 37°C for 30 min at 150 rpm. Digested homogenates were filtered through a 70-μM strainer to make single cell suspensions. Single cell suspensions were centrifuged at 50 × *g* for 3 min at 4°C to remove hepatocytes and undigested material. The supernatant was centrifuged at 500 × *g* for 10 min, and the pellet was resuspended in 20 ml ammonium chloride potassium lysis buffer for 5 min at 25°C to lyse the remaining red blood cells. After 2 washes in HBSS containing 0.1% bovine serum albumin (BSA) and 0.5 mM EDTA (flow buffer), the cells were counted with a hemocytometer. Cells (2.5 × 10^5^) were blocked with α-CD16/32 antibody (BioLegend; no. 101302) for 15 min on ice. After one wash with flow buffer, cells were incubated with violet live/dead stain (Thermo Fisher L34963) and ∼0.25 μg/ml antibodies for 30 min on ice to stain for cells of interest (α-CD45 [BioLegend; no. 103132], α-CD11b [BioLegend; no. 101226], α-Ly6G [BioLegend no. 127606]). To ensure that equal volumes of sample were analyzed, counting beads (Fisher Scientific; no. NC0318024) were used to normalize cell counts. Cells were subsequently washed twice with flow buffer and analyzed on a BD FACSCanto II instrument. Data were analyzed via the FlowJo software package version 16.0 (FlowJo, LLC).

### Immunofluorescence.

Organs were harvested and fixed in ice-cold PBS containing 4% paraformaldehyde for 18 to 24 h with gentle end-over-end mixing. Fixed organs were placed in 30% sucrose solution overnight. Saturated organs were then submerged in optimal cutting temperature compound (OCT) (Sakura) and flash-frozen in cassettes submerged in 2-methylbutane chilled with dry ice. Sections (10 μm) were permeabilized and stained with ∼1 μg/ml rabbit anti-myeloperoxidase (Abcam; no. ab9535), and 5 μg/ml rat anti-mouse CD68 (Thermo Fisher Scientific; no. 14-0681-82), followed by incubation with goat anti-rabbit AF594 (Thermo Fisher Scientific; no. A11012) and goat anti-rat AF488 (Thermo Fisher Scientific; no. A11006). Nuclei were visualized using mounting medium containing DAPI (4′,6-diamidino-2-phenylindole; Thermo Fisher Scientific). Sections were mounted on glass slides under no. 1.5 coverslips. Images were acquired with an inverted Zeiss LSM 880 confocal microscope with FAST AiryScan, using either a 10× Plan-Apochromat 0.45 NA objective or a 40× LD LCI Plan-Apochromat 1.2 NA immersion objective as indicated in the figure legend. Images were processed using the in-line AiryScan processing module in Zen Black.

### Intravital microscopy.

Mice were anesthetized with isoflurane and were kept on a 37°C heating pad throughout the experiment. All images were taken with a Leica SP8 upright confocal DM600 CFS confocal microscope equipped with a resonant scanner via a cover slip-corrected ×25, 0.95 NA water immersion objective (Leica Microsystems, Buffalo Grove, IL). In the sterile injury experiments, mice were injected retro-orbitally with 5 μl (2.5 μg) Ly6G-AF647 antibody (Clone 2A8; BioLegend, San Diego, CA) in 100 μl sterile Ca^2+^/Mg^2+^-free PBS (Gibco, Thermo Fisher Scientific, USA). The abdominal wall was opened with a transverse scission along the costal margin, and the left liver lobe was immobilized against a coverslip with a suction ring (53). Around the suction ring, the wound was covered with sterile wet gauze to mitigate desiccation. An area in the sinusoids with little motion artifact was chosen, and a focal burn injury was applied via 2-s-long illumination with a high-power two-photon laser beam on an ∼250 μm^2^ area. An ∼40,000 μm^2^ area with the burned injury centered was imaged in GFP and AF647 channels over 3 h in a time-serial, z-stack scanning mode. In septic injury experiments, the left femoral artery was cannulated with a PE-10 tube and the left liver lobe was exteriorized for confocal imaging as described above. The mouse was injected through the femoral artery cannula with 5 μl (2.5 μg) Ly6G-AF647 (BioLegend; no. 127610) in 100 μl of PBS. Four fields of view with low motion artifact and centered on the sinusoids were selected. Imaging was conducted for 3 h in GFP and Ly6G-AF647 channels in a multiposition, time-serial, z-stack scanning mode. Immediately following imagining initiation, 5 × 10^7^ CFU of GFP expressing S. aureus (described above) suspended in 100 μl Ca^2+^/Mg^2+^-free PBS were injected into the femoral artery cannula. Videos were analyzed for neutrophil and S. aureus accumulation with Imaris software (Bitplane, Concord, MA). Neutrophils were tracked via applying area modeling on the AF647 signal. Only neutrophils present in 3 consecutive frames were counted. S. aureus was counted via applying spot modeling on the GFP signal.

### Systemic chemical perfusions.

*In vivo* biotinylation was conducted as reported (13). Briefly, animals were anesthetized using isoflurane in a closed chamber, and a median sternotomy was performed. The left ventricle of the heart was punctured with a 25-gauge butterfly needle (BD Vacutainer) and a small cut was made in the right atrium to allow draining of perfusion solutions. All perfusion reagents were ice-cold and were infused using a perfusion pump (Fisher Scientific). Blood components were quickly washed out with PBS for 5 min at a rate of 5 ml/min. A solution containing 100 mM EZ-link sulfo-NHS-biotin (Thermo Fisher) in PBS, pH 7.4 was used to perfuse the animals at 3 ml/min for 10 min. Finally, animals were perfused with the quenching solution (50 mM Tris-HCl, pH 7.4) at 3 ml/min for 5 min. Control animals were perfused in the same way but with PBS.

### Organ preparations.

After *in vivo* biotinylation, mouse livers were harvested as previously described (13). Briefly, livers were harvested and homogenized using zirconia/silica beads (1 mm diameter; Biospec) in a benchtop MagNA Lyser instrument (Roche). Homogenization buffer contained 5 M urea, 0.25 M NaCl, and 0.1% SDS. Samples were briefly centrifuged at 16,100 × *g* for 5 min to sediment insoluble tissue debris. The clear supernatant was transferred to a new tube and further purified by centrifugation through a 0.45-μm filter (Costar/Millipore Sigma; no. CLS8163). Filtrate was collected, and protein concentration in the filtrate was quantified by bicinchoninic acid (BCA) assay (Thermo Scientific) per the manufacturer’s instructions and stored at −80°C until further analysis.

### Purification of biotinylated proteins.

After organ preparation, biotinylated proteins were purified as previously described (13). Briefly, biotinylated proteins were purified from homogenized liver (1 mg protein) using a Bravo AssayMap platform and AssayMap streptavidin cartridges (Agilent). Briefly, cartridges were prewashed with 50 mM ammonium bicarbonate (pH 8), and then samples were loaded. Nonbiotinylated proteins were removed by extensively washing the cartridges with 8 M urea in 50 mM ammonium bicarbonate buffer (pH 8). Cartridges were washed with rapid digestion buffer (Promega; rapid digestion buffer kit), and bound proteins were subjected to on-column digestion using mass spectrometry-grade trypsin/Lys-C rapid digestion enzyme (Promega, Madison, WI) at 70°C for 2 h. Released peptides were desalted in the Bravo platform using AssayMap C_18_ cartridges, and the organic solvent was removed by vacuum centrifugation (SpeedVac). Samples were stored at −20°C prior to liquid chromatography tandem mass spectrometry (LC–MS/MS) SWATH (sequential window acquisition of all theoretical fragment ion spectra) analysis.

### LC-MS/MS analysis.

Peptide analysis was performed on a Q Exactive HF-X mass spectrometer (Thermo Fisher Scientific) connected to an EASY-nLC 1200 ultra-high-performance liquid chromatography system (Thermo Fisher Scientific). Peptides were trapped on precolumn (PepMap100 C_18_ 3 μm; 75 μm by 2 cm; Thermo Fisher Scientific) and separated on an EASY-Spray column (ES803, column temperature 45°C; Thermo Fisher Scientific). Equilibrations of columns and sample loading were performed per the manufacturer’s guidelines. Solvent A was used as stationary phase (0.1% formic acid), and solvent B (mobile phase; 0.1% formic acid, 80% acetonitrile) was used to run a linear gradient from 5% to 38% over 120 min at a flow rate of 350 nl/min. The 44 variable window data-independent acquisition (DIA) method is described in detail in reference 54. Briefly, the mass range for MS1 was 350 to 1,650 *m/z* with a resolution of 120,000 and a resolution of 30,000 for MS2 with a stepped normalized collision energy (NCE) of 25.5, 27, and 30. The MS2 windows were 350 to 371, 370 to 387, 386 to 403, 402 to 416, 415 to 427, 426 to 439, 438 to 451, 450 to 462, 461 to 472, 471 to 483, 482 to 494, 493 to 505, 504 to 515, 514 to 525, 524 to 537, 536 to 548, 547 to 557, 556 to 568, 567 to 580, 579 to 591, 590 to 603, 602 to 614, 613 to 626, 625 to 638, 637 to 651, 650 to 664, 663 to 677, 676 to 690, 689 to 704, 703 to 719, 718 to 735, 734 to 753, 752 to 771, 770 to 790, 789 to 811, 810 to 832, 831 to 857, 856 to 884, 883 to 916, 915 to 955, 954 to 997, 996 to 1,057, 1,056 to 1,135, and 1,134 to 1,650 *m/z*.

### DIA data analysis.

An *in silico* spectral library was generated for the reference proteome of Mus musculus (EMBL-EBI RELEASE 2020_04; 22,295 entries) with a deep neural network from DIA-NN v1.7.10 (55). For search space reduction, a list of previously MS-detectible mouse peptides was compiled from multiple sources—Peptide Atlas (56) and the mouse spectral libraries from references 57 and 58, 265,780 entries. The generated library with 667,455 precursors was used for DIA data extraction with DIA-NN version 1.7.10 with a protein q-value of 0.01 and RT-profiling enabled.

### Statistical analysis.

For proteomics analysis, the statistical functions module from the Python package SciPy was used to identify proteins that were differentially abundant between sets of samples (using Student’s *t* test). Functional enrichment analysis of differentially abundant proteins was performed through the Database for Annotation, Visualization, and Integrated Discovery (DAVID). DAVID was run using default settings with the thresholds count ≥ 2 and EASE ≤ 0.1. Visualization of enriched Genome Ontology (GO) terms was produced using code adapted for treemap visualizations from the Web tool REVIGO. All other statistical analyses were performed in GraphPad Prism version 8, with a *P* value of < 0.05 considered statistically significant. Unless otherwise indicated, normally distributed pairwise comparisons were performed using a two-tailed Student’s *t* test, and nonnormal distributions were compared using the Mann-Whitney U test.

### Supplemental material.

Fig. S1 details BUN levels across multiple time points in sepsis in *Ndst1^f/f^Tie2Cre* mice and liver damage and infection parameters in *Ndst1^f/f^LysM;PF4Cre* mice. Fig. S2 highlights flow cytometry parameters and additional cell population counts from infected livers, as well as complete blood count analysis. 
