# Supplementary figures and images for: Endothelial Heparan Sulfate Mediates Hepatic Neutrophil Trafficking and Injury during Staphylococcus aureus Sepsis
Source: mBio. 2021 Sep 21;12(5):e01181-21. doi: 10.1128/mBio.01181-21 (PMC8546592; doi:10.1128/mBio.01181-21)

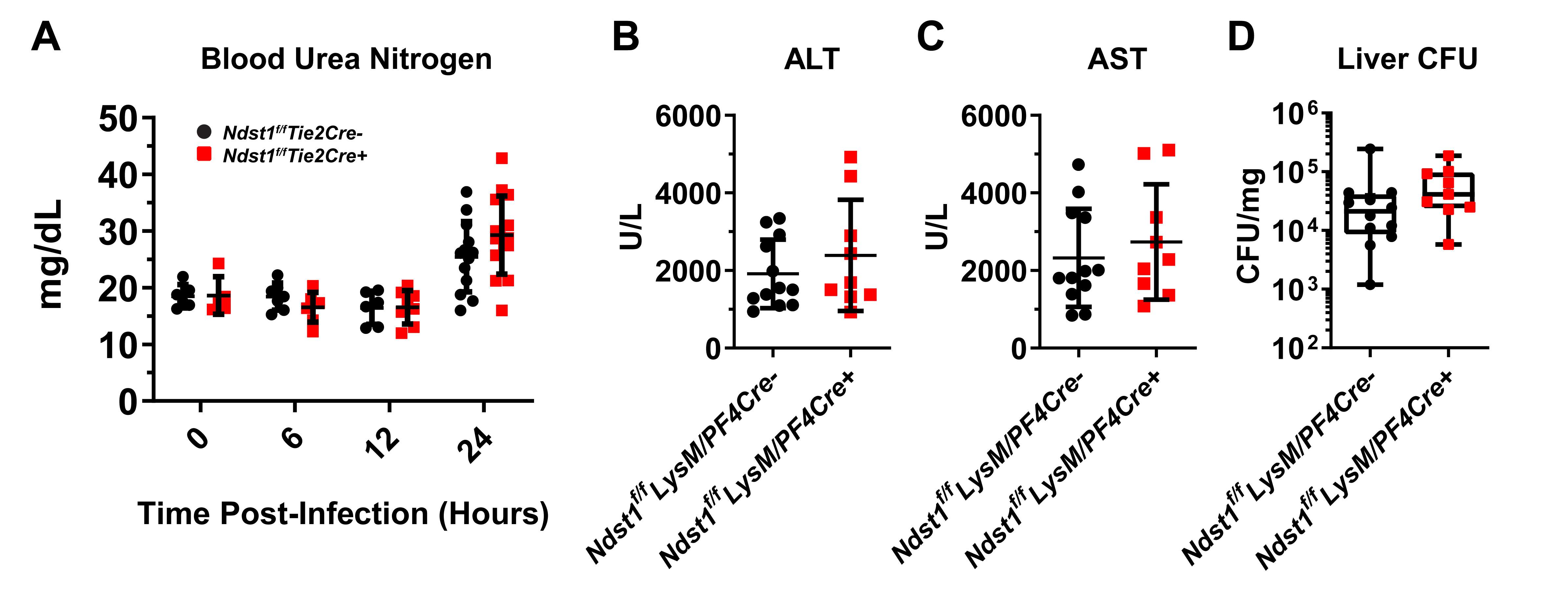

Supplement: FIG S1 [file mbio.01181-21-sf001.jpg]

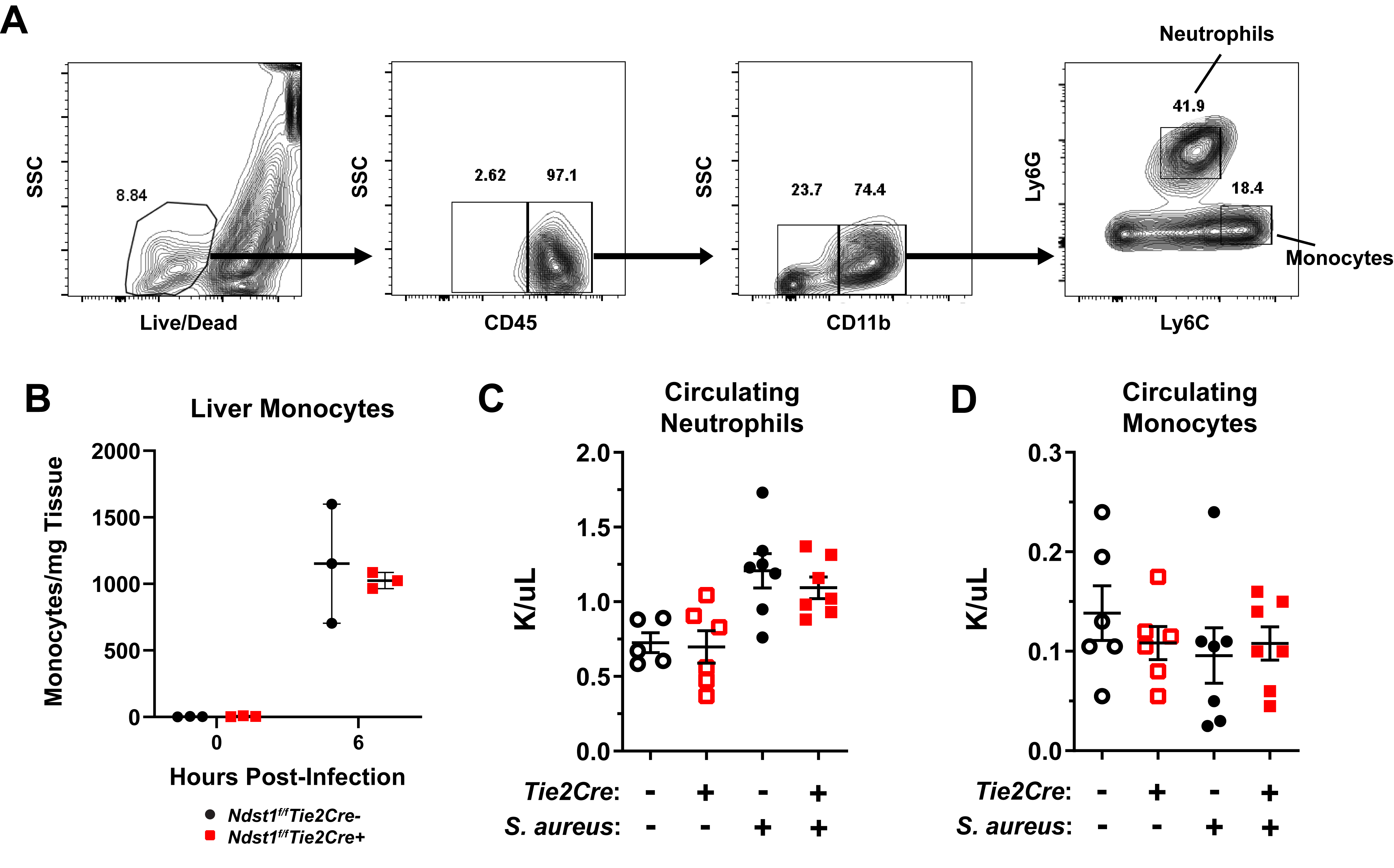

Supplement: FIG S2 [file mbio.01181-21-sf002.jpg]
